# Is There Still a Role for Autologous Stem Cell Transplantation in Older Adults with AML?

**DOI:** 10.3390/hematolrep18040057

**Published:** 2026-08-12

**Authors:** Saveria Capria, Clara Minotti, Massimo Breccia, Maria Laura Bisegna, Ida Carmosino, Patrizia Chiusolo, Luca Di Marino, Giovanni Luzi, Sabrina Mariani, Giovanni Marsili, Leonardo Marabitti, Elettra Ortu La Barbera, Lorena Scollo, Maurizio Martelli, Silvia Maria Trisolini

**Affiliations:** 1 Department of Hematology, AOU Policlinico Umberto I—Sapienza University, 00161 Rome, Italy; minotti@bce.uniroma1.it (C.M.); carmosino@bce.uniroma1.it (I.C.); trisolini@bce.uniroma1.it (S.M.T.); 2 Hematology, Department of Translational and Precision Medicine, Sapienza University, 00161 Rome, Italy; breccia@bce.uniroma1.it (M.B.); m.laurabisegna@gmail.com (M.L.B.); marabittileonardo@gmail.com (L.M.); lorena.scollo@uniroma1.it (L.S.); martelli@bce.uniroma1.it (M.M.); 3 Ematologia e Trapianto, Dipartimento di Scienze Ematologiche e di Laboratorio, Fondazione Policlinico Universitario A. Gemelli IRCCS, Sezione di Ematologia, Università Cattolica del Sacro Cuore, 00168 Rome, Italy; patrizia.chiusolo@unicatt.it (P.C.); luca.dimarinomd@gmail.com (L.D.M.); 4 UOC Ematologia e Trapianto di Cellule Staminali, San Camillo-Forlanini Hospital, 00152 Rome, Italy; 1970giovanni@gmail.com; 5 Hematology Unit, AOU Sant’Andrea, Sapienza University, 00189 Rome, Italy; sa.mariani@uniroma1.it; 6 GIMEMA Foundation, 00182 Rome, Italy; g.marsili@gimema.it; 7 Department of Hematology, SM Goretti Hospital, University Polo Pontino, 04100 Latina, Italy

**Keywords:** acute myeloid leukemia, autologous stem cell transplantation, elderly

## Abstract

**Introduction**: Autologous stem cell transplantation (ASCT) is regaining interest as a consolidation strategy for acute myeloid leukemia (AML) patients with favorable/intermediate risk and measurable residual disease (MRD) negativity. In fit older adults, its role remains debated due to concerns regarding toxicity and stem cell collection. Aim: To evaluate the long-term outcomes of ASCT in AML patients aged ≥60 years in first complete remission (CR1). **Methods**: We retrospectively analyzed 43 non-M3 AML patients (median age 64, range 60–72) who underwent ASCT in CR1 across five centers (2000–2024). Risk was stratified by ELN 2017. Primary endpoints were overall survival (OS) and disease-free survival (DFS). **Results**: The cohort included 22 favorable-risk and 21 intermediate-risk patients. Most (88%) achieved CR1 after one induction course. Median neutrophil and platelet engraftment occurred at 13 and 15 days, respectively. Grade III/IV infections were observed in 28% of patients; transplant-related mortality was low at 4%. At a median follow-up of 77 months, 5-year OS and DFS were 51% (95% CI: 37–70%) and 47% (95% CI: 34–65%), respectively. OS was significantly superior in favorable- versus intermediate-risk patients (68% vs. 35%, *p* = 0.027), with a similar trend for DFS (57% vs. 35%, *p* = 0.07). The number of consolidation cycles did not impact survival outcomes. **Conclusions**: This multicenter study confirms that ASCT is a feasible and effective consolidation strategy for selected fit AML patients aged ≥60. The low mortality and encouraging survival, particularly in favorable-risk subgroups, support ASCT as a valid alternative to prolonged chemotherapy or maintenance.

## 1. Introduction

Acute myeloid leukemia (AML) is predominantly a disease of older adults, with a median age at diagnosis of approximately 67 years. Outcomes in this population have historically been poor because of the high prevalence of adverse cytogenetic and molecular features, antecedent hematologic disorders, and reduced tolerance to intensive chemotherapy and allogeneic hematopoietic stem cell transplantation (allo-HSCT) [1].

Despite these limitations, intensive chemotherapy (IC) remains a feasible therapeutic option in carefully selected older patients. In fit individuals without major comorbidities, IC can improve overall survival (OS) and response rates even in those aged ≥70 years, particularly among patients with low- to intermediate-risk disease who achieve measurable residual disease (MRD) negativity after induction and consolidation [2].

In patients with high-risk AML, the role of allo-HSCT as post-remission therapy is well established. This has become especially relevant in the current era, in which less toxic regimens—such as hypomethylating agents combined with targeted therapies—can induce high complete remission rates and serve as a bridge to allogeneic transplantation [3].

By contrast, the optimal management of patients with favorable/low-risk or intermediate-risk AML, as well as those who are not candidates for allo-HSCT but achieve complete remission, remains uncertain. In particular, defining the most appropriate post-remission strategy for fit older patients continues to represent an unmet clinical need.

Several prognostic models are available to stratify risk and predict outcomes after induction therapy in older individuals. An effective therapeutic framework for this population should integrate measures of treatment tolerability and expected benefit, using accurate prognostic stratification to guide the use of intensive approaches while balancing the risks of toxicity and treatment-related mortality [4,5].

Historically, intensive treatment followed by consolidation with autologous stem cell transplantation (ASCT) in fit older patients has been limited by stem cell mobilization and collection failure, early relapse, and patient refusal. Recently, however, this strategy has regained interest in patients with favorable or intermediate prognoses and MRD negativity, and it should be evaluated against other contemporary post-remission approaches currently available for this selected population [6].

In this multicenter study, we retrospectively assessed the long-term outcomes of older AML patients undergoing ASCT in CR1 across five Italian centers, providing real-world data to inform current clinical practice.

## 2. Materials and Methods

This study was conducted in accordance with the principles of the Declaration of Helsinki. Data were collected anonymously using pre-specified variables. All patients provided informed consent for the collection and use of their medical data at the time of transplantation, in line with EBMT guidelines.

We retrospectively reviewed 43 consecutive patients with newly diagnosed AML (non-M3) aged 60 years or older who underwent ASCT in CR1 at five hematology centers between 2000 and 2024. Only patients with complete molecular characterization at the time of diagnosis were included. Data were manually extracted from medical records using a unified case report form across all centers, including demographics, molecular findings, treatment details, and clinical outcomes.

Risk stratification was performed according to ELN 2017 guidelines, because the cohort spans 2000–2024, and ELN 2017 was the most consistently applicable framework across the dataset [7]. Patients were considered fit for IC/ASCT based on physician assessment and institutional practice. First-line chemotherapy consisted of a 3 + 7 regimen ± etoposide [8] until 2016 and 3 + 7 ± gemtuzumab ozogamicin since 2017 [9]. Patients deemed ineligible for anthracycline treatment received high-dose cytarabine and fludarabine (FLAG regimen) [10]. Patients achieving complete remission (CR) or CR with incomplete hematologic recovery (CRi) underwent intermediate- or high-dose cytarabine-based consolidation. Stem cell mobilization and collection were planned after hematologic recovery from the first consolidation cycle.

Pre-transplant MRD was assessed, within 30 days before conditioning, on bone marrow samples by RT-qPCR in patients with NPM1 mutation [11], RUNX1–RUNX1T1, or CBFB–MYH11 rearrangements. In patients lacking a suitable molecular marker, a multiparameter flow cytometry assay with a threshold of at least 10–4 was employed [12].

Conditioning regimens were administered according to each center’s policy (Table 1). The majority of patients received busulphan-based regimens (BU-CY: busulphan 3.2 mg/kg + cyclophosphamide 120 mg/kg—Bu-Mel: busulphan 3.2 mg/kg + melphalan 140 mg/m^2^—Bu-VP16: busulphan 3.2 mg/kg + etoposide 60 mg/kg), or the BEAM/FEAM regimen (carmustine or fothemustine 300 mg/m^2^, ara-c 800 mg/m^2^, etoposide 800 mg/m^2^, melphalan 140 mg/m^2^). Antimicrobial prophylaxis followed institutional standards. Growth factor support (G-CSF) was used at the treating physician’s discretion.

The primary endpoints were overall survival (OS) and disease-free survival (DFS). OS was defined as the time (months) from ASCT to the last follow-up for survivors or death from any cause. DFS was defined as the time (months) from ASCT to the last follow-up for survivors or the occurrence of relapse, progression, or death from any cause.

Secondary endpoints included non-relapse mortality (NRM), engraftment kinetics, non-hematologic toxicities, and baseline/treatment variables potentially associated with DFS. NRM was defined as death after ASCT not attributable to relapse or disease progression. Toxicities were graded according to the Common Terminology Criteria for Adverse Events (CTCAE), version 4.03.

OS and DFS were estimated using the Kaplan–Meier method, and survival distributions were compared with the log-rank test. The reverse Kaplan–Meier method was used to estimate the median follow-up duration. Cumulative incidence of relapse was estimated using the cumulative incidence function, with non-relapse mortality considered as a competing event. Categorical variables were compared using the chi-square test; continuous variables were summarized as median (range) and compared using the Mann–Whitney U test. A two-tailed *p*-value < 0.05 was considered statistically significant.

All analyses and data visualization were generated using the statistical computing environment R (4.4.2 R Core Team, R Foundation for Statistical Computing, Vienna, Austria).

## 3. Results

Forty-three patients (22 females, 21 males) were included. Median age at transplantation was 64 years (range, 60–72), and 21 patients (49%) were aged ≥65 years. Baseline molecular features are reported in Table 1.

Twenty-two patients had favorable-risk disease, and 21 had intermediate-risk disease.

Most patients (88%) received one induction course. Consolidation therapy consisted of one cycle in 28 patients and two cycles in 15 patients. The median time from diagnosis to ASCT was 5 months (range, 2–17).

Engraftment occurred at a median of 13 days for neutrophils (range, 9–35) and 15 days for platelets (range, 10–58). Grade III–IV infections occurred in 12 patients (28%), in detail: two pneumonia, one urinary tract infection, four CMV reactivation, and five sepsis. Grade III/IV extra-hematological toxicity was 4% (one typhlitis, one acute renal failure). Overall NRM rate was 4% (one sepsis, one GI hemorrhage).

At a median follow-up of 77 months (IQR, 39–119), 19 patients remained in CR (44%), 22 relapsed (51%, median time to relapse, 8 months; range, 1–108), and 2 died from transplant-related causes at day +36 and +39. Among favorable-risk patients, five received an allo-HSCT after relapse; at the last follow-up, three are alive, and two died from NRM.

**Figure 1:**
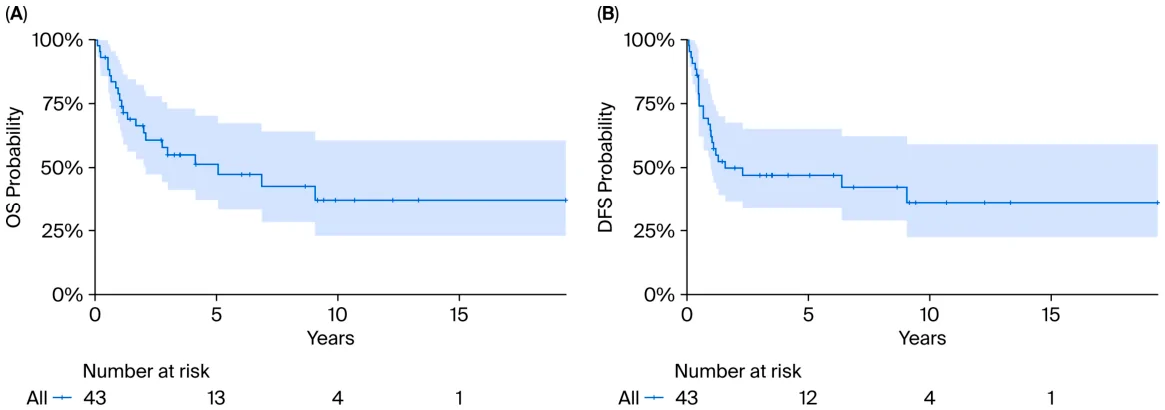
(**A**) Overall survival and (**B**) disease-free survival of AML patients undergoing ASCT.

The 5-year estimated OS for the overall cohort was 51% (95% CI, 37–70%), with a median OS of 61 months. The 5-year estimated DFS was 47% (95% CI, 34–65%), with a median DFS of 19 months (Figure 1). Overall cumulative incidence of relapse at 1 year was 33% (CI 19%, 48%), while NRM estimated at 100 days and 1 year was 5% (CI 1%, 14%).

**Figure 2:**
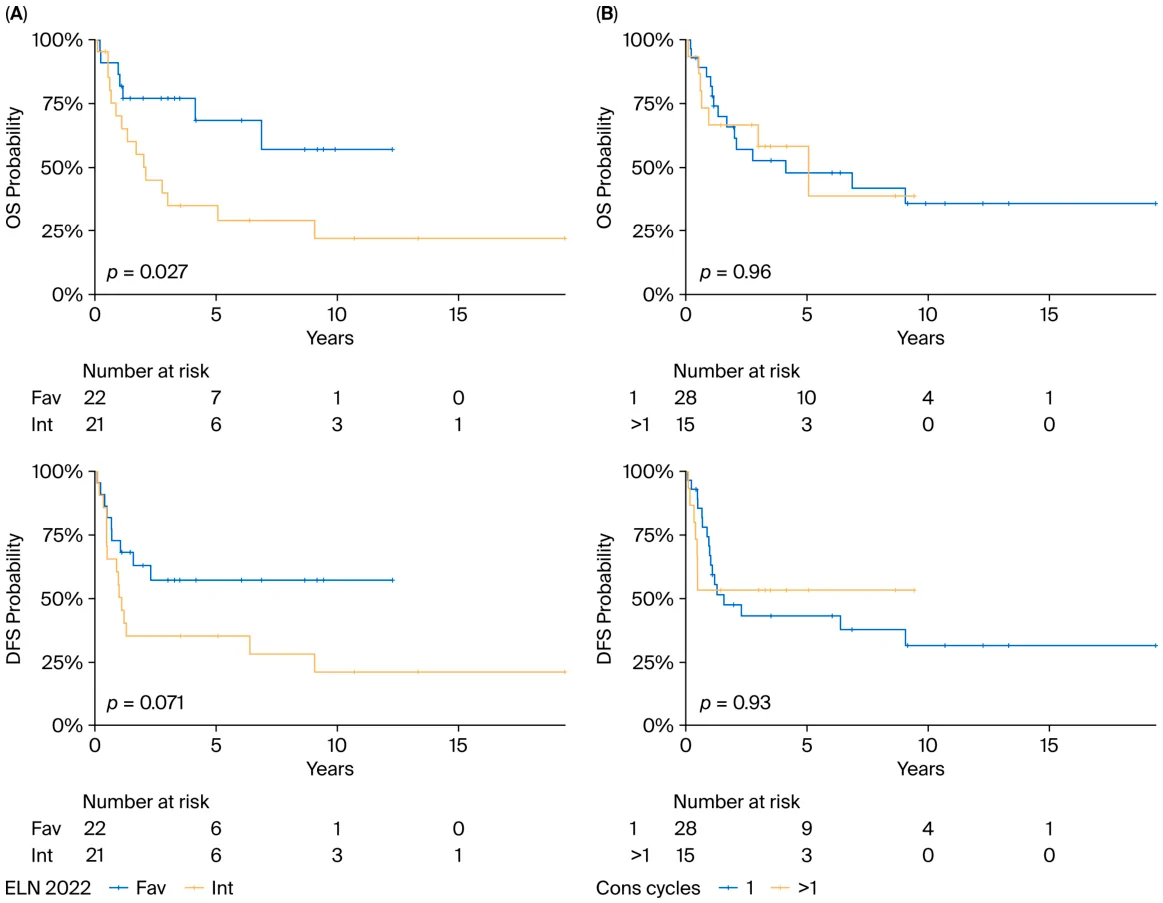
(**A**) Overall survival and disease-free survival by ELN risk. (**B**) Overall survival and disease-free survival by number of consolidation cycles.

In univariate analysis, 5-year OS was significantly higher in favorable- versus intermediate-risk patients: 68% (95% CI, 49–95%) vs. 35% (95% CI, 19–64%), *p* = 0.027. Five-year DFS was 57% (95% CI, 39–83%) vs. 35% (95% CI, 19–64%), showing a trend favoring favorable-risk patients without reaching statistical significance (*p* = 0.07). In the favorable-risk subgroup, median OS and DFS were not reached at 5 years post-transplant (Figure 2A).

The number of consolidation cycles was not associated with better survival. As shown in Figure 2B, when comparing patients receiving one versus >1 consolidation cycle, 5-year OS estimates were nearly overlapping: 48% (95% CI, 32–73%) vs. 58% (95% CI, 37–91%) (*p* = 0.96). Similarly, DFS at 5 years was 43% (95% CI, 28–67%) vs. 53% (95% CI, 33–86%) (*p* = 0.93).

**Figure 3:**
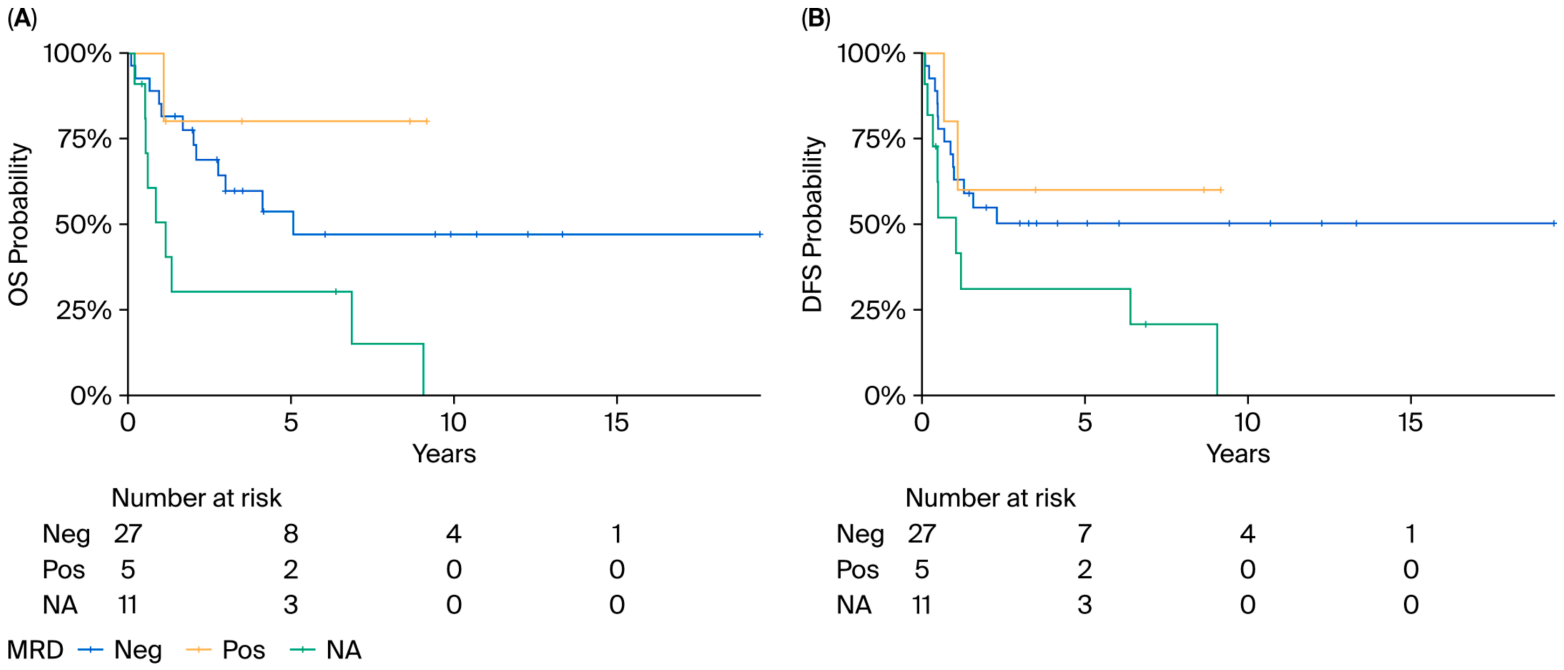
(**A**) Overall survival and (**B**) disease-free survival by MRD pre-ASCT.

Pre-transplant MRD data were available for 32/43 patients; MRD was assessed by molecular methods in nine cases and by flow cytometry in 23 cases. Overall, 27 patients were MRD-negative, and 5 were MRD-positive. Five-year OS and DFS estimates according to MRD status are shown in Figure 3. Given the small sample size, we preferred not to perform a statistical test in this patients’ subset.

## 4. Discussion

The treatment of AML in older adults remains a major unmet clinical need due to the high prevalence of adverse biological features—clonal hematopoiesis and epigenetic dysregulation-inducing mutations—and to the frequency of comorbidities that may limit eligibility for intensive chemotherapy [2,4].

For fit patients with intermediate- to high-risk AML, allo-HSCT remains the primary curative option, requiring rigorous assessment of comorbidities, performance status, cognitive function, and social support. While post-induction allo-HSCT rates in CR1 are historically around 18% [13], venetoclax-hypomethylating agent combinations yield 60–70% response rates, facilitating transplantation in selected patients aged 60–74. However, substantial transplant-related mortality (29% at 1 year) necessitates careful candidate selection to optimize survival benefits [3].

The American Society of Hematology 2025 guidelines for the treatment of newly diagnosed AML in older adults emphasize that conventional induction and post-remission therapy may be preferred for patients with favorable-risk cytogenetic or molecular features and for those in whom prolonged hospitalization is acceptable. In addition, “younger older adults” (e.g., patients in their 60 s) may tolerate conventional induction and post-remission chemotherapy better than older age groups. For patients who achieve remission after at least one cycle of conventional induction and are not candidates for allo-HSCT, the guidelines recommend post-remission therapy rather than observation alone [14].

However, optimal post-remission strategies for fit older adults who are not candidates for allo-HSCT remain poorly defined.

Autologous peripheral blood stem cell transplantation is gaining renewed interest as a component of AML management of adults with favorable/intermediate-risk disease and post-consolidation MRD negativity, with the aim to avoid the toxicity of allogeneic transplantation in patients who may already be cured.

Evidence supporting autologous transplantation versus chemotherapy alone dates back to randomized trials from the 1990s and subsequent prospective cooperative studies (HOVON/SAKK). In younger adults, ASCT is associated with improved DFS compared with chemotherapy alone, without a consistent OS advantage. These findings have been influenced by the feasibility of salvage allo-HSCT in CR2 and were generated largely before the widespread adoption of MRD-guided strategies and contemporary risk stratification [15,16].

MRD assessment is now widely recognized as a key tool for refining baseline risk classification. According to ELN recommendations, one of the most informative time points is after two cycles of intensive chemotherapy (MRD2), which increasingly informs therapeutic decision-making due to its strong prognostic value. The GIMEMA MRD-driven strategy for intermediate-risk AML in patients ≤ 60 years uses post-consolidation MRD to allocate eligible patients to autologous or allogeneic transplantation; results from the prospective AML1310 trial showed comparable outcomes in MRD-positive patients receiving allogeneic transplantation and MRD-negative patients undergoing autologous transplantation [17]. Data from the ongoing GIMEMA AML 1819 trial (NCT04168502), applying a similar MRD-oriented strategy, will further clarify the role of this approach also within favorable-risk diseases.

In older patients, prospective evaluation of ASCT has historically been limited by factors such as inadequate stem cell collection, early relapse, refusal, and toxicity concerns. However, advances in mobilization strategies, transplantation techniques, and supportive care have substantially reduced the impact of these limitations [6].

In our real-world multicenter cohort of fit AML patients aged ≥60 years undergoing ASCT in CR1, transplantation was feasible and generally well tolerated. Median OS and DFS for the entire cohort were 61 and 19 months, respectively, with a low NRM of 5%. Long-term outcomes were particularly encouraging among favorable-risk patients, in whom median OS and DFS were not reached at 5 years. Although the sample size precluded more granular stratification by MRD status, survival curves suggested sustained long-term outcomes, with 5-year OS and DFS estimates of 68% and 57% in the favorable-risk subgroup.

One notable finding was that the number of post-remission consolidation cycles beyond the first did not appear to influence long-term outcomes. This suggests that continuing conventional chemotherapy rather than proceeding promptly to high-dose consolidation may be unnecessary once disease burden has been cleared. Prospective evaluation is warranted, especially in standard-risk patients who fail to achieve MRD-negative remission after two cycles. A practical advantage of this approach is a limited overall treatment duration (approximately 5–6 months), potentially allowing most patients to resume normal activities while avoiding prolonged outpatient monitoring and repeated cyclical therapies.

Recently, oral maintenance strategies have expanded the therapeutic landscape for non-HSCT candidates. In FLT3-ITD–mutated AML, the Quantum-FIRST trial demonstrated improved outcomes with quizartinib plus chemotherapy across induction, consolidation, and maintenance for patients up to age 75, including those skipping first-line transplantation [18,19].

For older patients with intermediate- to high-risk de novo or secondary AML in CR1 ineligible for HSCT, oral azacitidine (CC-486) was approved based on the QUAZAR AML-001 trial. Despite improving overall and relapse-free survival (median OS: 24.7 months), long-term survival remained limited (<30% at 5 years) with frequent relapse-driven discontinuations. Furthermore, CC-486 caused significant gastrointestinal toxicity and myelosuppression—often requiring dose modifications—alongside a 4% fatal infectious or hemorrhagic event rate [19].

These data raise an important question in the current therapeutic era: how should clinicians best treat fit older patients in whom a potentially curative approach is intended? For high-risk disease or MRD-positive intermediate-risk disease in fit candidates for allo-HSCT, allogeneic transplantation remains the preferred option, supported by recent evidence enabling less intensive bridging strategies [3]. However, in fit patients who are not candidates for allo-HSCT due to biological features or comorbidities—and in whom a chronic monthly strategy with limited survival benefit (as reported in VIALE-A [20]) is not desirable—the key decision lies between intensive consolidation followed by maintenance and an intensive high-dose approach consolidated by ASCT. In our institution, the therapeutic strategy is largely driven by measurable residual disease (MRD) status. We aim to offer intensive consolidation followed by autologous stem cell transplantation (ASCT) to patients who have a concrete probability of cure with this approach. In this regard, pivotal insights will be provided by the forthcoming data from the GIMEMA AML 1819 clinical trial, which is currently nearing completion.

A limitation of our study lies in the potential risk of selection bias. Given its retrospective and multicenter nature, information is lacking on the number of patients in complete remission who did not proceed to transplantation due to mobilization failure or treatment-related toxicity. Furthermore, our study lacks a control group to which our results can be compared.

In conclusion, in the context of increasing life expectancy, careful assessment of fitness and biological risk are crucial to identify patients most likely to benefit from ASCT in terms of durable disease control and long-term outcome. Overall, this multicenter real-world analysis supports ASCT as a feasible and effective strategy in selected AML patients aged ≥60 years, with encouraging survival and minimal NRM. Although retrospective, these findings suggest that ASCT warrants reconsideration in prospective studies, potentially integrated with novel targeted therapies.

## Figures and Tables

**Table 1:** Baseline characteristics and treatment details of the study cohort. (Bu-CY = busulphan/cyclophosphamide. Bu-based = busulphan/melphalan, busulphan/etoposide. BEAM = carmustine, ara-c, etoposide, melphalan. FEAM = fothemustine, ara-c, etoposide, melphalan).

Patients’ Details		No. (%)
Total pts (M/F)		43 (21/22)
Median age ys. (range)		64 (60–72)
	<65 ys.	22 (51%)
	>65 ys.	21 (49%)
ELN risk	favorable	22 (51%)
	intermediate	21 (49%)
Molecular marker	NPM1	12 (28%)
	CBFβ-MYH11	7 (16%)
	RUNX1-RUNX1T1	4 (9%)
	FLT3-ITD	1 (2%)
	KMT2A	1 (2%)
	No molecular marker	18 (42%)
No. of consolidation cycles	1 cycle	28 (65%)
	>1 cycle	15 (35%
Conditioning regimen	Bu-Cy/Bu-based	11/20 (72%)
	BEAM/FEAM	2/5 (16%)
	other	5 (12%)

## Data Availability

The data are available from the medical records upon reasonable request.
